# Special Issue: Adenosine Receptors

**DOI:** 10.3390/molecules22071220

**Published:** 2017-07-20

**Authors:** Francisco Ciruela, Eddy Sotelo

**Affiliations:** 1 Unitat de Farmacologia, Departament Patologia i Terapèutica Experimental, Facultat de Medicina, IDIBELL-Universitat de Barcelona, L’Hospitalet de Llobregat, 08907 Barcelona, Spain; 2 Institut de Neurociències, Universitat de Barcelona, 08035 Barcelona, Spain; 3 Centro Singular de Investigación en Química Biolóxica e Materiais Moleculares (CIQUS), Universidad de Santiago de Compostela, 15782 Santiago de Compostela, Spain; e.sotelo@usc.es; 4 Departamento de Química Orgánica, Facultad de Farmacia, Universidade de Santiago de Compostela, 15782 Santiago de Compostela, Spain

Nearly 90 years ago, Drury and Szent-Györgyi revealed that adenosine produced profound hypotension and bradycardia, and it affected kidney function in mammals [1]. Since then, the physiological role and potential therapeutic use of adenosine have been extensively explored [2,3]. Adenosine is considered a retaliatory metabolite [4], thus increasing oxygen supply and decreasing oxygen consumption. Consequently, this purine nucleoside can potentially regulate a large amount of physiological processes, including respiratory regulation, hormone action, neural function, platelet aggregation, lymphocyte differentiation, and vascular tone. In addition, adenosine exerts a negative chronotropic and dromotropic effect on the heart, as well as it mediates the inhibition of neurotransmitters release and lipolysis. Interestingly, while adenosine can promote coronary artery dilatation [5], it can induce small renal blood vessels contraction, thus opposite effects (i.e., vasodilatation vs. vasoconstriction, respectively) could be observed depending on the physiological territory studied [6]. Overall, it has been assumed that this purine nucleoside is a mediator of metabolic distress, thus having considerable impact on homeostatic cellular functioning.

In 1972, it was demonstrated that electrical stimulation of brain slices prompted adenosine release [7]. Importantly, this released adenosine induced an increase in intracellular cAMP levels, a phenomenon that was blocked by methylxanthines (i.e., caffeine and theophylline) [8,9]. Indeed, these observations constituted the first piece of evidence pointing to the existence of specific plasma membrane adenosine receptors (ARs). Conversely, it was also demonstrated that the adenosine-mediated antilipolytic effect occurred with a concomitant reduction in cAMP [10]. Overall, this dual effect of adenosine on cAMP formation ended with the first subclassification of ARs into A_1_ and A_2_ [11], which were assumed to be G protein-coupled receptors (GPCRs). Interestingly, four subtypes of ARs have been identified so far, namely A_1_R, A_2A_R, A_2B_R, and A_3_R, showing differential pharmacological profile, tissue distribution, and effector coupling [12]. 

The following special issue of *Molecules* is designed to summarizes the main aspects of the state of the art of ARs, paradigm of purinergic GPCRs, providing to the reader with the recent developments in ARs ligands and their potential therapeutic utility. Accordingly, four review articles highlight the pharmacotherapeutic usefulness of ARs selective agonists and antagonists. Geldenhuys et al. review the role of adenosine in cardiovascular function and discuss these ARs ligands that have been identified so far, modulating the cardiovascular adenosinergic system [13]. Thus, the authors emphasize on how small changes in ligand structure impact ligand potency and efficacy [13]. Therefore, considering the importance of adenosine signaling in the cardiovascular system, ARs represent a valuable class of drug targets with extraordinary therapeutic potential to treat people with high blood pressure and related cardiovascular disorders. Interestingly, Sousa and Diniz further review the current development ARs-based drugs in the therapeutic of vasculature-related diseases [14]. Thus, the authors highlight the renewed interest in developing ARs ligands for cardiovascular pathologies (i.e., hypertension, heart failure and stroke), which is reflected by the increased number of recent patents and clinical trials involving the adenosinergic system in the pharmacotherapy of cardiovascular diseases [14]. In the Central Nervous System (CNS), adenosine has been shown to play a key regulatory role, acting as a presynaptic, postsynaptic, and/or non-synaptic neuromodulator [15]. Extracellular adenosine in the brain is related to the intracellular concentration of adenosine and nucleotides, such as ATP, AMP, and cAMP [16]. Interestingly, in some brain areas like the striatum, it has been proposed that the main source of extracellular adenosine is the intracellular cAMP [17]. Thus, striatal extracellular adenosine would mostly reflect an increased activation of receptors positively linked to adenylyl cyclase. Stockwell et al. discuss the role of CNS A_1_R and A_2A_R in both normal and pathological conditions, and they outline the potential therapeutic use of these receptors in neurodegenerative diseases (i.e., Parkinson’s disease, PD) [18]. In addition, the authors highlight the cross-talk between A_1_R and A_2A_R, supported by the existence A_1_R/A_2A_R heteromers [19], and its role in several neurodegenerative disorders, including ischemia, stroke, epilepsy, and PD [18]. Finally, Ciancetta and Jacobson review the structure-based molecular modeling applied to elucidate the binding of agonists to the A_3_R. The authors discussed the challenges associated with an accurate prediction of the receptor extracellular vestibule through homology modeling from the available X-ray templates [20]. Indeed, A_3_R agonists have emerged as promising antinociceptive agents, as well as for the treatment of rheumatoid arthritis, psoriasis, and hepatocellular carcinoma [20].

Subsequently, four research articles assess new ARs functional, mechanistic, and medicinal chemistry prospects. Thus, Arin et al. evaluate the expression of A_2B_R and adenosine deaminase (ADA) in rabbit gastric mucosa enterochromaffin-like (ECL) cells [21]. The authors identify A_2B_R and ADA at the cell surface of histamine-producing ECL cells originating from the body of the rabbit stomach, which upon A_2B_R challenge the receptor couples to Gs protein. Collectively, these results shed light into the role of adenosine in the physiology of histamine secretion in ECL cells [21]. Indeed, this is an important physiological discovery considering the elevated local adenosine levels found during inflammation of the gastric mucosa, which may blow histamine production in ECL cells and contribute to modulate acid secretion. Next, Zsuga et al. provide an in silico qualitative method assessing receptor reserve for adenosine [22]. Adenosine is an endogenous agonist with a short half-life because of its high exposure to enzymes and transporters, thus it is difficult to determine a reliable value for receptor reserve. Hence, the authors describe a new method to determine receptor reserve for adenosine, transferable to other short half-live endogenous agonists, which may allow prediction of agonist efficacy in different tissues based on the relevant receptor reserve values [22]. Subsequently, Deganutti and Moro use an alternative computational method, the Supervised Molecular Dynamics (SuMD) approach, to investigate the ligand-receptor recognition pathway on a nanoseconds time scale [23]. Thus, to verify the applicability of the methodology, the authors select two fragment-like adenosineA_2A_R positive allosteric modulators (PAMs) and explore their possible recognition pathways by performing SuMD simulations in the absence or presence of an agonist (i.e., NECA) [23]. Interestingly, the results suggest that SuMD simulations can allow the identification of fragment-like PAMs following their receptor recognition pathways, characterizing the possible allosteric binding sites. Finally, Fernández-Dueñas et al. describe the synthesis of a novel bivalent ligand consisting in the covalent linking of caffeine and docosahexaenoic acid (DHA) [24]. Indeed, the authors assessed the pharmacological activity and possible toxicity of caffeine-DHA in a simple cellular model [24]. Caffeine, a non-selective AR antagonist, is the most consumed psychoactive substance worldwide. It is generally used because it enhances physical and cognitive functions, improving alertness, physical performance, and concentration, while decreasing fatigue [25]. In addition, it has been postulated that this natural substance may have beneficial effects in neurodegenerative diseases such as PD [26], thus behaving as an inverse agonist [27]. DHA is the major omega-3 fatty acid present in the mammalian brain, where it is involved in many essential processes such as neurogenesis and neurotransmission. The rationale for attaching caffeine to DHA is to facilitate caffeine delivery to striatal A_2A_Rs and to increase the rate of A_2A_R oligomerization with dopamine D_2_ receptor in the striatum [28].

Overall, we hope that this timely focused issue summarizing our current knowledge on adenosine receptors will be of interest to a wide range of readers of the journal interested in the purinergic field. Finally, we wish to express our best thanks to all authors and co-authors of the issue for their commitment and to the anonymous reviewers for their excellent contributions.
